# Genetic Evidence Supporting a Causal Role of Snoring in Erectile Dysfunction

**DOI:** 10.3389/fendo.2022.896369

**Published:** 2022-05-25

**Authors:** Yang Xiong, Xin Zhong, Fuxun Zhang, Wei Wang, Yangchang Zhang, Changjing Wu, Feng Qin, Jiuhong Yuan

**Affiliations:** ^1^ Andrology Laboratory, West China Hospital, Sichuan University, Chengdu, China; ^2^ Department of Urology, West China Hospital, Sichuan University, Chengdu, China; ^3^ Department of Epidemiology and Health Statistics, School of Public Health and Management, Chongqing Medical University, Chongqing, China

**Keywords:** snoring, erectile dysfunction, causal estimates, Mendelian randomization, genetic evidence

## Abstract

**Background:**

The association between snoring and erectile dysfunction (ED) is inconsistent in multiple observational studies. To clarify the causal association of snoring on ED, we performed this two-sample Mendelian randomization study.

**Materials and Methods:**

The single nucleotide polymorphisms (SNPs) associated with snoring were retrieved from the UK biobank cohort with 314,449 participants (117,812 cases and 196,637 controls). The summary statistics of ED were obtained from the European ancestry with 223,805 subjects (6,175 cases and 217,630 controls). Single-variable Mendelian randomization (MR) and multivariable MR were used to assess the causal relationship between snoring and ED.

**Results:**

Snoring increases the risk of ED (Odds ratio [OR] = 3.45, 95% confidence interval [CI] = 1.68 - 7.09, *P* < 0.001) in the inverse variance weighting estimator. In sensitivity analyses, the ORs for the weighted median, MR robust adjusted profile score, and MR Pleiotropy Residual Sum and Outlier approach, MR-Egger, and maximum likelihood method are 5.70 (95% CI = 1.19 - 27.21, *P* < 0.05), 3.14 (95% CI = 1.01 - 9.72, *P* < 0.05), 3.11 (95% CI = 1.63 - 5.91, *P* < 0.01), 1.23 (95% CI = 0.01 – 679.73, *P* > 0.05), and 3.59 (95% CI = 1.07 – 12.00, *P* < 0.05), respectively. No heterogeneity and pleiotropy are observed (*P* for MR-Egger intercept = 0.748; *P* for global test = 0.997; *P* for Cochran’s Q statistics > 0.05). After adjusting for total cholesterol, triglyceride, low-density lipoprotein, and cigarette consumption, the ORs for ED are 5.75 (95% CI = 1.80 - 18.34, *P* < 0.01), 4.16 (95% CI = 1.10 - 15.81, *P* < 0.05), 5.50 (95% CI = 1.62 - 18.69, *P* < 0.01), and 2.74 (95% CI = 1.06 - 7.10, *P* < 0.05), respectively.

**Conclusion:**

This study provides genetic evidence supporting the causal role of snoring in ED.

## Introduction

According to the definition of the National Institutes of Health (NIH) Consensus Development Panel on Impotence, erectile dysfunction (ED) refers to the inability to attain or maintain the penile erection, leading to unsatisfactory sexual intercourse (1). ED remains prevalent globally (2). As indicated by two key epidemiological surveys, the prevalence of ED ranges from 5% to 15% between 40-70 years in the Massachusetts Male Aging Study and on average, that is 30% across different age groups in the European Male Ageing Study (3, 4). It has been disclosed that ED patients have higher risks of depression, impaired self-esteem, infertility, stroke, and sub-clinical cardiovascular disease, which heavily burdens the males and further requires effective interventions to attenuate the high prevalence (5–8).

Multiple psychogenic or organic associated factors like depression, anxiety, and diabetes have been identified in the onset of ED (9). Of them, snoring is noted to be involved with ED (10). Snoring, a sort of sleep-disordered breathing, is a feature for obstructive sleep apnea (OSA), which commonly coexists with ED (11). However, the role of OSA is inconsistent across different literature. As indicated by Kalejaiye O et al. (12), males with OSA had significantly lower erectile function than the control group. According to the findings from Hanak V et al. (13), the sexual satisfaction domain score for the snorers was lower than that for the none/mild snorers. But, the levels of erectile function remained similar across different snoring categories. Additionally, in other previous studies exploring the association between OSA and ED, the results fluctuated markedly when adjusting different covariates, and even further yield opposite conclusions (14, 15). The discrepancy may be attributed to the limited sample size, cross-sectional design, and especially confounding factors. These defects cannot be overcome by the observational design and new method, such as Mendelian randomization (MR), is required to clarify the inconsistent findings, which are still absent.

MR is an epidemiological method using genetic variants to produce causal inference (16). The genetic variants are single nucleotide polymorphisms (SNPs) identified by genome-wide association studies (GWAS). When forming a zygote, the SNPs are assorted randomly regardless of postnatal confounding factors like diabetes, obesity, and hypertension (17). The random distributed SNPs are used as instrumental variables (IVs) to replace the exposures (i.e. snoring) and outcomes (i.e. ED). Therefore, a naturally formed randomized controlled trial (RCT) is imitated, which avoids reverse causality and biases from confounders, and then yields causal estimates (17). To date, no study has clarified the causal links between snoring and ED. To address this concern, we use the conventional single-variable MR (SVMR) and multivariable MR (MVMR) design to explore whether snoring is causally involved with the onset of ED.

## Materials and Methods

### Data Sources of Snoring, ED, and Adjusted Covariates

The genetic estimates for snoring were derived from the white British ancestry extracted from the Neale Lab (https://gwas.mrcieu.ac.uk/datasets/ukb-a-14/). The snoring cohort included 314,449 subjects (117,812 cases and 196,637 controls). Participants were defined as a snorer according to the positive answer to a touchscreen question: “Does your partner or a close relative or friend complain about your snoring?” (18). Missing data and uninformative reports like “I don’ know” and “Prefer not to answer” were excluded from the cohort.

The summary level dataset of ED was retrieved from one previous GWAS study in the European ancestry (19). By incorporating three cohorts, the combined cohort enrolled 223,805 subjects (6,175 cases and 217,630 controls), which had stronger inference power. The diagnosis of ED was based on the International Classification of Diseases version 10 (ICD-10) codes (N48.4 and F52.2), or a medical intervention history for ED like surgery or oral drugs, or self-report from the participants. Detailed information regarding the two phenotypes can be further accessed through previous publications (18, 19).

Summary-level statistics of the adjusted covariates including the low-density lipoprotein (LDL), cigarette consumption, total cholesterol (TC), and triglyceride (TG) were retrieved from previous studies (20, 21). After excluding the participants from 23&Me project, a sample size of 1.2 million individuals were subjected to detect the genetic estimates of cigarette consumption (20). To obtain the genetic estimates of lipid levels, the European population genotyped with GWAS arrays4 or Metabochip array were enrolled into analyses. A total of 188,577 participants were included (21). Detailed information regarding the ancestry, sample sizes, consortium, etc. were described in **Supplemental Table 1**.

### Instrument Variable Selection

To identify SNPs closely associated with snoring, genetic instruments with genome-wide statistical significance ≥ 5 × 10^-8^ were filtered. Further, to identify the independent SNPs assorted randomly during gestation, the left SNPs were then subjected to the calculation of linkage disequilibrium (LD) using the PLINK clumping approach. The LD was calculated based on 1000 Genomics European reference panel. SNPs with LD *r^2^* ≥ 0.001 at a window size of 10,000 Kb were pruned. Additionally, the MR-Steiger filtering was applied to calculate the variance explained in the exposure (i.e. snoring) and the outcome (i.e. ED) and further test whether the variance explained in snoring is significantly higher than that in ED or not (22). The insignificant results indicate a reverse causal direction that the extracted SNPs may primarily affect the ED than the snoring, which should be removed. In this study, all the extracted SNPs passed the test and were not excluded. Besides, the palindromic SNPs were deleted from the extracted SNPs.

To avoid the bias from weak instrumental variables (IVs), *F*-statistics were calculated, using the following formula: *F*-statistics = (Beta/Se) ^2^. *F*-statistics represent the strength of IVs and the mean of *F*-statistics was calculated as the overall statistics. Generally, *F*-statistics > 10 were set as the threshold of strong IVs. The *F*-statistics of all the extracted SNPs were > 10. Therefore, no SNPs were excluded in this step. Moreover, to reduce the heterogeneity and avoid the pleiotropy, radial-MR and MR Pleiotropy Residual Sum and Outlier (MR-PRESSO) methods were used to identify the horizontal pleiotropic outliers (23, 24). No outliers were detected in this study. All the left SNPs were available in the outcome dataset. Hence, no proxy SNP was used in the MR analyses. Finally, 19 SNPs were left and used as IVs in this study. The detailed information of the IVs is displayed in **Table 1**.

**Table 1:** SNPs used as genetic instruments in the Mendelian randomization analyses.

SNP	Chr	Position	A1	A2	BETA	SE	P	Palindromic	F	Gene
rs10062026	5	90052289	A	G	0.006976	0.001246	2.14E-08	FALSE	31.36	ADGRV1
rs10505911	12	24022160	A	C	-0.00796	0.001446	3.72E-08	FALSE	30.29	SOX5
rs10878271	12	65795603	C	T	0.007551	0.001244	1.28E-09	FALSE	36.84	MSRB3
rs11075985	16	53805207	A	C	-0.00738	0.001212	1.11E-09	FALSE	37.13	FTO
rs1108431	16	31054607	T	C	-0.00805	0.001237	7.61E-11	FALSE	42.36	STX4
rs12925525	16	1773914	T	G	-0.01341	0.002392	2.07E-08	FALSE	31.43	MAPK8IP3
rs13251292	8	71474355	G	A	-0.00803	0.001222	4.97E-11	FALSE	43.19	TRAM1
rs1641511	17	7559677	A	G	0.007981	0.001413	1.62E-08	FALSE	31.9	ATP1B2
rs1775550	10	9052742	A	G	0.009168	0.001533	2.21E-09	FALSE	35.78	RP11-428L9.2
rs199497	17	44866602	C	T	-0.01043	0.001648	2.48E-10	FALSE	40.05	WNT3
rs2307111	5	75003678	C	T	0.007038	0.001226	9.52E-09	FALSE	32.94	POC5
rs2614464	14	99743113	A	G	0.007823	0.001212	1.10E-10	FALSE	41.64	BCL11B
rs34811474	4	25408838	A	G	0.008518	0.001416	1.81E-09	FALSE	36.17	ANAPC4
rs592333	13	51340315	G	A	-0.00896	0.001204	1.01E-13	FALSE	55.36	DLEU7
rs61597598	2	1.57E+08	A	G	-0.01212	0.001752	4.63E-12	FALSE	47.84	LINC01876
rs62066451	17	46316540	G	A	0.016689	0.002789	2.17E-09	FALSE	35.81	SKAP1
rs7930256	11	88849434	C	T	-0.00705	0.00125	1.70E-08	FALSE	31.81	AP001482.1
rs9309771	3	77593064	G	A	0.007558	0.001202	3.22E-10	FALSE	39.54	ROBO2
rs9515311	13	1.12E+08	T	C	0.006995	0.001238	1.63E-08	FALSE	31.9	ANKRD10

### Statistical Analyses

To obtain the total and direct causal estimates between snoring and ED, the SVMR, and MVMR were performed. The causal estimates were assessed using the inverse variance weighting (IVW) approach. The IVW method uses the meta-analysis technique to combine the effects from individual IVs to an overall weighted effect (25). When all the SNPs are valid IVs, this approach can produce consistent estimation, which is considered the main result in MR analyses.

The inclusion of more IVs can increase the statistical power in MR analyses, which yet may introduce the pleiotropic SNPs into analyses. The pleiotropy refers to the association between the IVs and other confounders except for the exposure (i.e. snoring in this study). This association can mediate the exposure-outcome links through other pathways, violating the basic assumption of MR and yielding biased estimates. To test and avoid the pleiotropy, several sensitivity analyses including MR-Egger, the weighed median, MR-PRESSO, and robust adjusted profile score (MR.RAPS) were performed.

MR-Egger method is adapted from Egger regression. This approach performs a weighted linear regression and introduces an intercept term into the regression function. As indicated by Bowden J (26), the MR-Egger estimator can produce unbiased estimates even when all the IVs are invalid IVs. Additionally, the distance between the introduced intercept term and zero can be adopted to quantify the directional pleiotropy. As for the weighted median estimator, it can yield consistent causal estimates when half of the IVs are valid (27). This method has greater precision in the estimates than the MR-Egger approach and better type 1 error rates than the IVW estimator. In this study, we also calculated MR.RAPS to assess the causal association between snoring and ED (28). By performing a linear model adjusting for the profile likelihood of the summary data, MR.RAPS can yield robust causal estimates. This estimator considers the weak IVs bias and remains consistent when weak IVs exist. As reported by Zhao et al., MR.RAPS displays higher statistical power than other conventional MR estimators and is statistically sound to both systematic and idiosyncratic pleiotropy (28).

Heterogeneity is another major concern in MR analyses, suggesting the possible concurrent presence of pleiotropy. To evaluate the heterogeneity between the IVs, IVW, MR-Egger, and Maximum likelihood methods were used. Cochran’s Q statistic was employed to quantify the heterogeneity. Further, we also adopted the leave-one-out analysis to identify the influential IVs on the estimates. This approach excluded one IV at a time and then performed MR analysis again using the IVW method.

The statistical power to detect the difference is evaluated using an online tool (https://shiny.cnsgenomics.com/mRnd/). Under the type I error rate of 0.05, the statistical power of snoring on ED is 100%. Moreover, the overlap and bias are calculated using an online software (https://sb452.shinyapps.io/overlap/). Under the type I error rate of 0.05 and assuming the overlap proportion is 100%, the value of bias is 0.026. This indicates that the overlap of the population is less likely to bias the finding.

Metabolisms are well-known risk factors for the occurrence of ED. And the snorers are usually correlated with disordered metabolisms. To obtain the direct effect from snoring, LDL, TC, TG and cigarette consumption were considered into further MVMR analyses. For the MVMR analyses, the overlapping SNPs between snoring and adjusted factors were used as the IVs. The IVW estimator was employed to yield the direct causal effect estimates after controlling the LDL, cigarette consumption, TC, and TG, respectively.

All the SVMR and MVMR analyses and relevant figures were made by R 3.6.5 (R Foundation for Statistical Computing, Vienna, Austria), using the “TwoSampleMR”, “RadialMR”, “mr.raps”, and “forestplot” packages. *P* < 0.05 (two-sided) is set as the significant threshold in statistics.

## Results

### Causal Effect Estimates of Snoring on ED in SVMR

**Figure 1:**
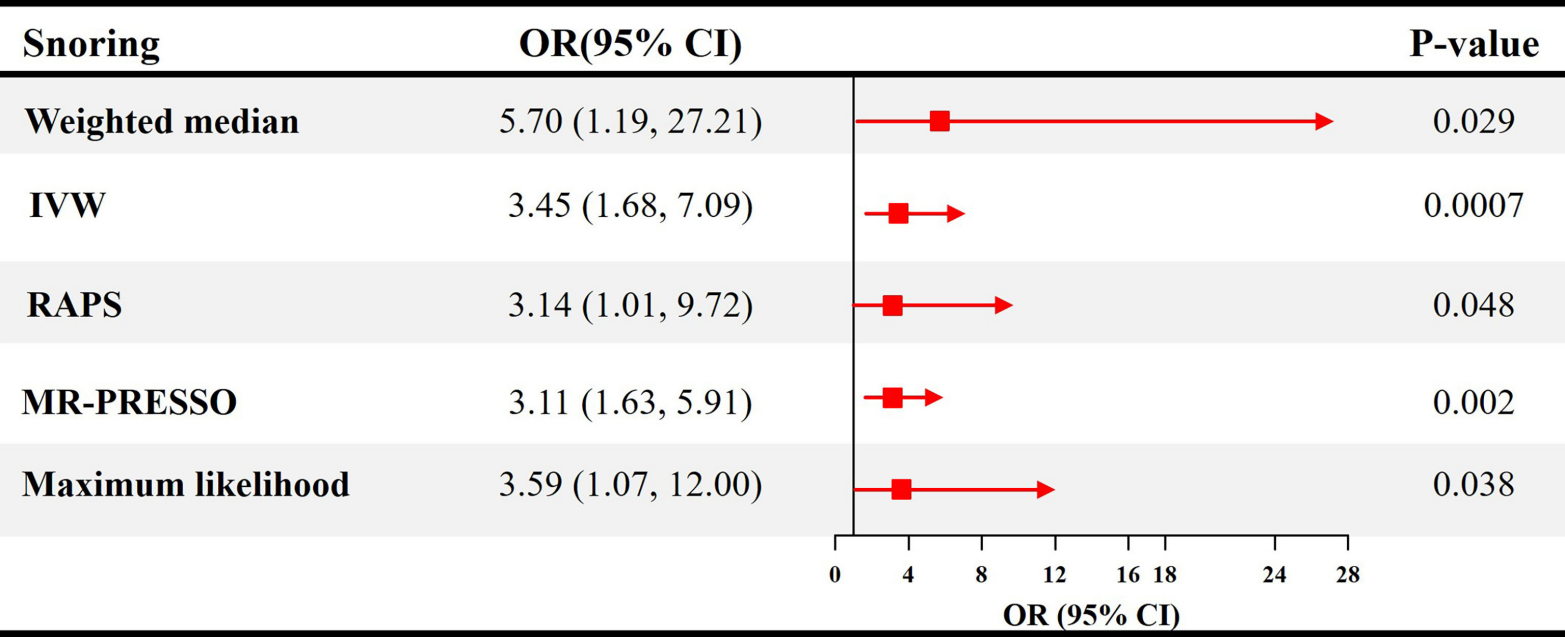
Causal estimates of snoring on ED in SVMR. OR, odds ratio; CI, confidence interval; IVW, Inverse variance weighted method; RAPS, robust adjusted profile score; MR, mendelian randomization; PRESSO, pleiotropy residual sum and outlier; ED, erectile dysfunction; SVMR, single-variable mendelian randomization.

**Figure 2:**
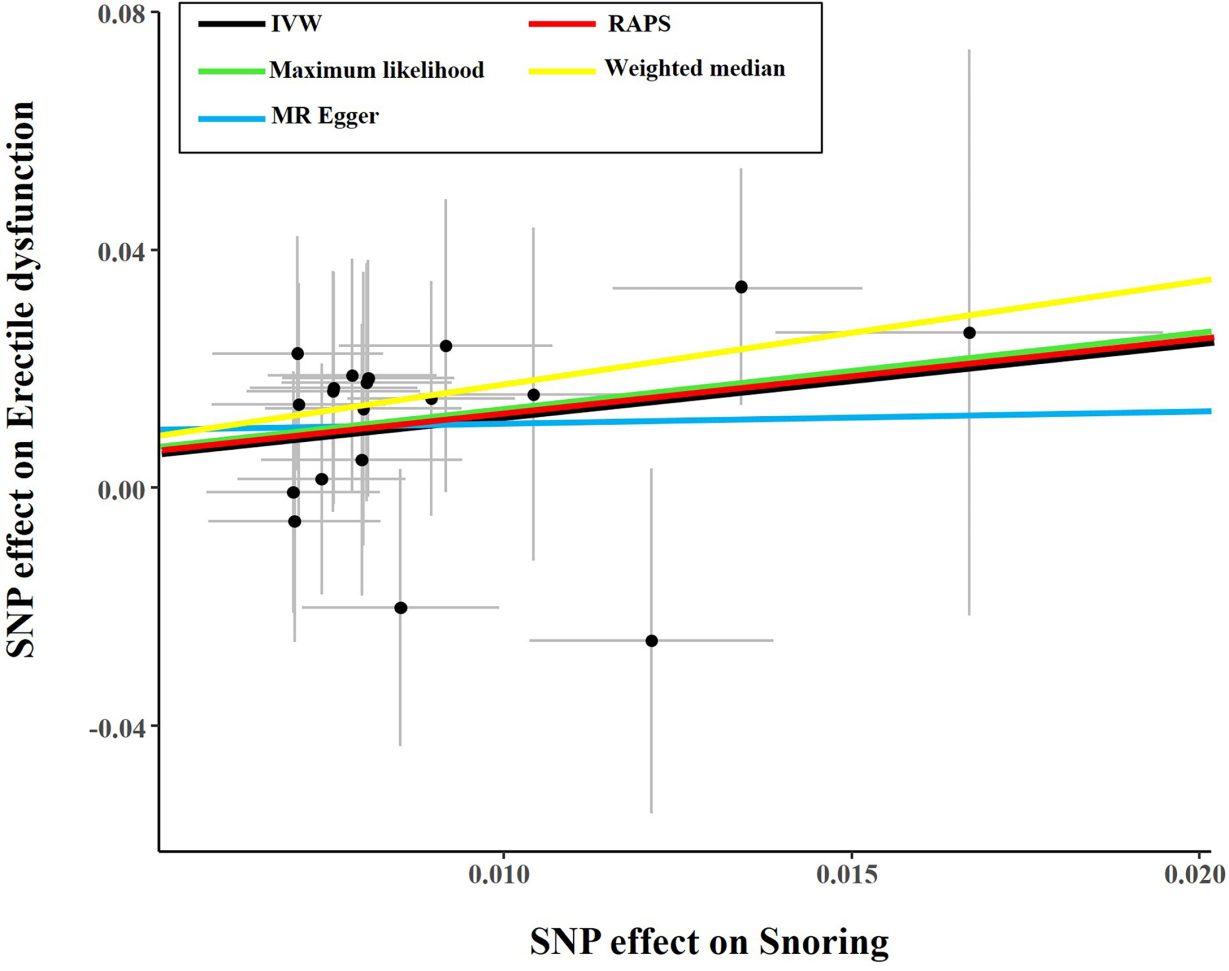
Scatter plot of the effect size of each SNP on snoring and ED in SVMR. SNP, single nucleotide polymorphism; IVW, Inverse variance weighted method; ED, erectile dysfunction; MR, mendelian randomization; SVMR, single-variable mendelian randomization.

The causal effect estimates of snoring on ED are displayed in **Figure 1**. In **Figure 1**, the IVW estimator reveals that snoring is associated with a 3.45-fold risk of ED (95% confidence interval [CI] = 1.68 - 7.09, *P* < 0.001). In sensitivity analyses, the odds ratios (ORs) for the weighted median, MR robust adjusted profile score, and MR Pleiotropy Residual Sum and Outlier approach, MR-Egger, and maximum likelihood method are 5.70 (95% CI = 1.19 - 27.21, *P* < 0.05), 3.14 (95% CI = 1.01 - 9.72, *P* < 0.05), 3.11 (95% CI = 1.63 - 5.91, *P* < 0.01), 1.23 (95% CI = 0.01 – 679.73, *P* > 0.05), and 3.59 (95% CI = 1.07 – 12.00, *P* < 0.05), respectively (**Figure 1** and **Table 2**). As indicated in **Figure 2**, with the increase of IVs’ effect on snoring, the risk of ED increases.

**Table 2:** MR estimates from each method of the causal effect of snoring on ED.

MR method	OR	95% CI	*P* value	Cochran's Q statistic	Heterogeneity *P* value	MR-Egger intercept	Intercept *P* value
MR-Egger	1.23	0.01 - 679.73	0.949	6.42	0.989	0.0087	0.748
IVW	3.45	1.68 - 7.09	0.0007	6.53	0.994	–	–
Maximum likelihood method	3.59	1.07 - 12.00	0.038	6.50	0.994	–	–

MR, Mendelian randomization; OR, odds ratio; SE, standard error; CI, confidence interval; ED, erectile dysfunction; IVW, Inverse variance weighted method.

Additionally, in **Table 2**, the MR-Egger test detects no directional pleiotropy (Intercept = 0.0087, *P* = 0.748). The MR-PRESSO test also finds no pleiotropy (Global test *P* = 0.997). The Cochran’s Q statistics are 6.42 (*P* = 0.989), 6.53 (*P* = 0.994), and 6.50 (*P* = 0.994) for the MR-Egger, IVW, and Maximum likelihood method, respectively (**Table 2**), suggesting the absence of heterogeneity. The funnel plot visualizing the heterogeneity is displayed in **Supplemental Figure 1**. The leave-one-out analysis identifies no influential IVs in the association between snoring and ED (**Supplemental Figure 2**). The estimates from each IV are visualized in **Supplemental Figure 3**.

### Causal Effect Estimates of Snoring on ED in MVMR

**Figure 3:**
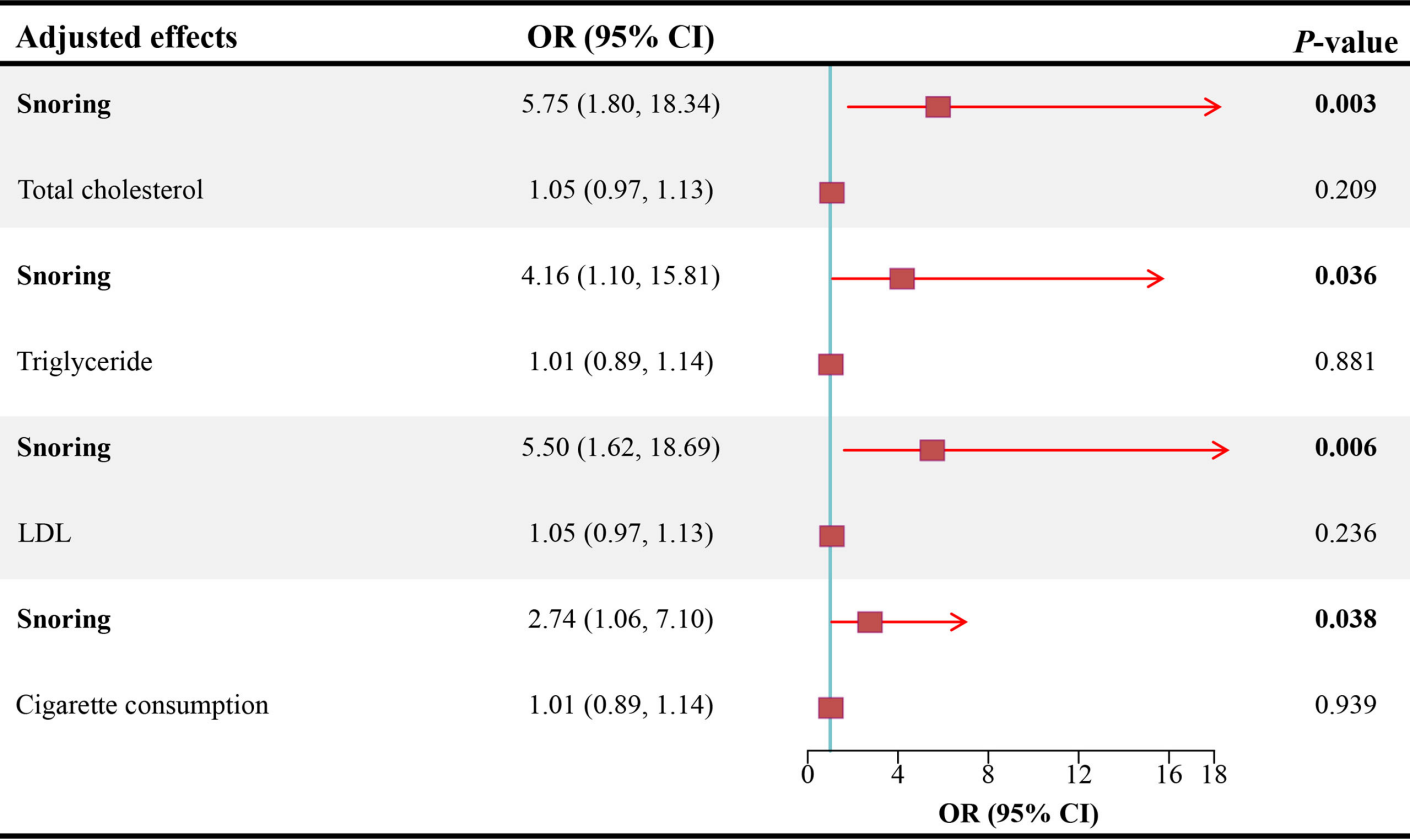
Causal estimates of snoring on ED in MVMR. OR, odds ratio; CI, confidence interval; LDL, low-density lipoprotein; ED, erectile dysfunction; MVMR, multivariable mendelian randomization.

**Figure 4:**
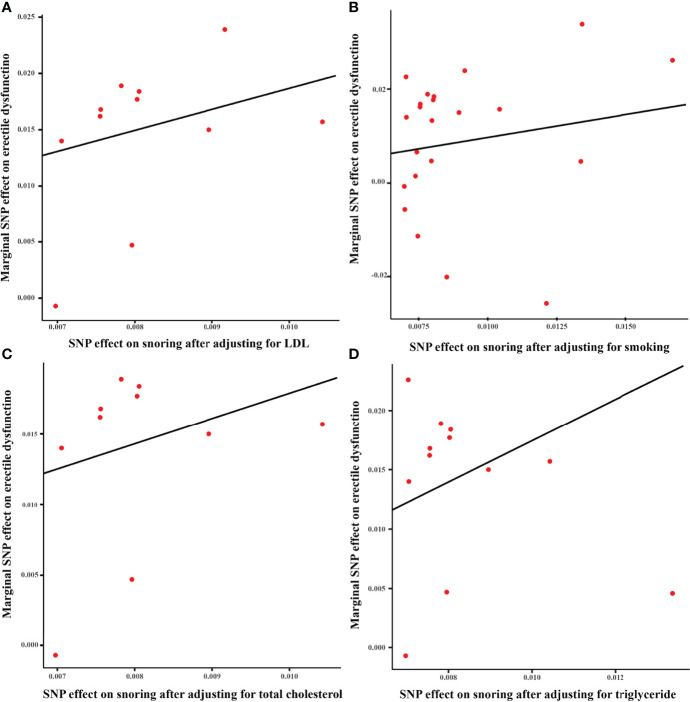
Scatter plot of the effect size of each SNP on snoring and ED in MVMR. **(A)** Scatter plot adjusting for LDL; **(B)** Scatter plot adjusting for smoking; **(C)** Scatter plot adjusting for total cholesterol; **(D)** Scatter plot adjusting for triglyceride. SNP, single nucleotide polymorphism; LDL, low-density lipoprotein; ED, erectile dysfunction; MVMR, multivariable mendelian randomization.

As shown in **Figure 3**, after adjusting for TC, TG, LDL, and cigarette consumption, the ORs for ED are 5.75 (95% CI = 1.80 - 18.34, *P* < 0.01), 4.16 (95% CI = 1.10 - 15.81, *P* < 0.05), 5.50 (95% CI = 1.62 - 18.69, *P* < 0.01), and 2.74 (95% CI = 1.06 - 7.10, *P* < 0.05), respectively. The scatter plots of the SNP-snoring association against SNP-ED association are shown in **Figure 4A** (controlling for LDL), **Figure 4B** (controlling for smoking), **Figure 4C** (controlling for TC), and **Figure 4D** (controlling for TG), respectively.

## Discussion

Given the absence of rigorously controlled clinical trials and longitudinal prospective studies, the inconsistent association of snoring on ED is hard to be clarified. Under the framework of MR design, this study provides causal evidence that the snorers have a higher risk of ED.

The loud snoring is an indicator of OSA, which has been found to be correlated with carotid atherosclerosis, coronary heart disease, and hypertension (11, 29, 30). The majority of the cross-sectional and case-control studies reported that OSA was negatively associated with erectile function. In a cross-sectional study with 467 participants, Andersen ML et al. (31) found that males with OSA had a 2.13-fold risk of ED than the controls. Similar findings are also reported by Heruti R et al. in Israelite (32). In the biggest case-control study, Petersen M et al. recruited 308 OSA cases and 1185 controls and disclosed that OSA patients had worse general and functional sexuality than the healthy counterparts. Besides the cross-sectional design, Chen et al. revealed a 9.44-fold risk of ED in the OSA patients than the control group in a longitudinal cohort enrolling 53,335 respondents (33).

Contrary to the findings stated above, there is accumulating evidence supporting the unrelated OSA in the suffering of ED. As disclosed by Bozorgmehri S et al. (34), males with higher apnea-hypopnea index displayed similar erectile function assessed by the 5-item International Index of Erectile Function. This cross-sectional study included 2,857 American men. Additionally, in the US, Hanak V et al. recruited 827 men using the stratified random sampling method and detected no association between OSA and ED. These findings were in line with one previous study (35). The inconsistent findings may be owing to the limited sample size, study design, and more importantly, the confounders, which are addressed in our study. In addition, only a few previous studies were prospective and defined the direction of causality. Our findings indicate that snoring increases the risk of erectile dysfunction, instead of the contrary. A clear causal direction facilitates guiding clinical decision-making.

Although the causal association of snoring on ED is established, the specific molecular mechanisms still need further exploration. Vascular endothelial dysfunction in OSA patients has been noted over the years, which may be responsible for linking OSA to ED. In OSA patients, sleep fragmentation and intermittent hypoxia trigger elastic fiber disruption, fiber disorganization, and reduced endothelial nitric oxide (NO) bioavailability of the blood vessel, indicating the impairment of vascular reactivity (36, 37). Of them, NO acts as a pivotal role in mediating the relaxation of penile blood vessels and cavernous smooth muscle through the NO/cGMP pathway (38). Consequently, reduced endothelial NO bioavailability harms the penis erection and leads to ED. In clinical studies, sildenafil, an inhibitor of cGMP degradation, can improve erectile function in OSA patients with ED, with higher satisfaction than continuous positive airway pressure (CPAP) (39). However, given the high proportion of dissatisfaction in both sildenafil and CPAP groups (50% versus 75%), the therapeutic benefits may be limited. This indicates that several other pathways may also involve in the onset of ED in OSA patients. Therapy targeting different pathways should be explored in future studies.

This study has some merits and shortcomings. The main merit is the MR framework, which overcomes the endogeneity and bias from confounding factors. Given the difficulties of RCT, this study paves the way for the prevention of impotence by targeting OSA. In addition, the included samples were confined to European descent, avoiding the population architecture bias but limiting the generalizability of our findings. Moreover, there may be a partial overlap in the samples of snoring and ED, possibly leading to the over-fitting of the models and undermining the causal inference power (40). However, given the usage of strong IVs (*F*-statistics > 10) in the analyses, the bias may be minimal. Besides, in light of the binary evaluation of snoring (snorers or controls) and the lack of individual statistics, the non-linear association between snoring and ED cannot be explored (40).

In conclusion, this study provides genetic evidence supporting a causal role of snoring in the onset of ED, independent of LDL, TC, TG, and cigarette consumption. Medical interventions should be considered for snorers to attenuate the high prevalence of ED.

## Conclusions

This study provides genetic evidence supporting a causal role of snoring in ED.

## Data Availability Statement

The original contributions presented in the study are included in the article/**Supplementary Material**. Further inquiries can be directed to the corresponding author.

## Ethics Statement

Ethical review and approval were not required for the study on human participants in accordance with the local legislation and institutional requirements. The patients/participants provided their written informed consent to participate in this study.

## Author Contributions

YX and XZ performed the data analyses and wrote the manuscript; WW, FZ, YZ, CW, and FQ revised the manuscript; JY participated in the study design and helped draft the manuscript. All authors contributed to the article and approved the submitted version.

## Funding

This work was supported by the Natural Science Foundation of China (81871147 & 82071639).

## Conflict of Interest

The authors declare that the research was conducted in the absence of any commercial or financial relationships that could be construed as a potential conflict of interest.

## Publisher’s Note

All claims expressed in this article are solely those of the authors and do not necessarily represent those of their affiliated organizations, or those of the publisher, the editors and the reviewers. Any product that may be evaluated in this article, or claim that may be made by its manufacturer, is not guaranteed or endorsed by the publisher.

## Supplementary Material

Supplementary Figure 1 The funnel plot in SVMR. IVW, Inverse variance weighted method; SVMR, single-variable mendelian randomization. IV, instrumental variable; SE, standard error; MR, mendelian randomization.

Supplementary Figure 2 The leave-one-out sensitivity analyses of snoring and ED. SNP, single nucleotide polymorphism; OR, odds ratio; CI, confidence interval; ED, erectile dysfunction; MR, mendelian randomization.

Supplementary Figure 3 The results of MR analyses of causal associations between each snoring SNP and ED. SNP, single nucleotide polymorphism; OR, odds ratio; CI, confidence interval; ED, erectile dysfunction; MR, mendelian randomization.
