# Supplementary figures and images for: Genetic Evidence Supporting a Causal Role of Snoring in Erectile Dysfunction
Source: Front Endocrinol (Lausanne). 2022 May 25;13:896369. doi: 10.3389/fendo.2022.896369 (PMC9174907; doi:10.3389/fendo.2022.896369)

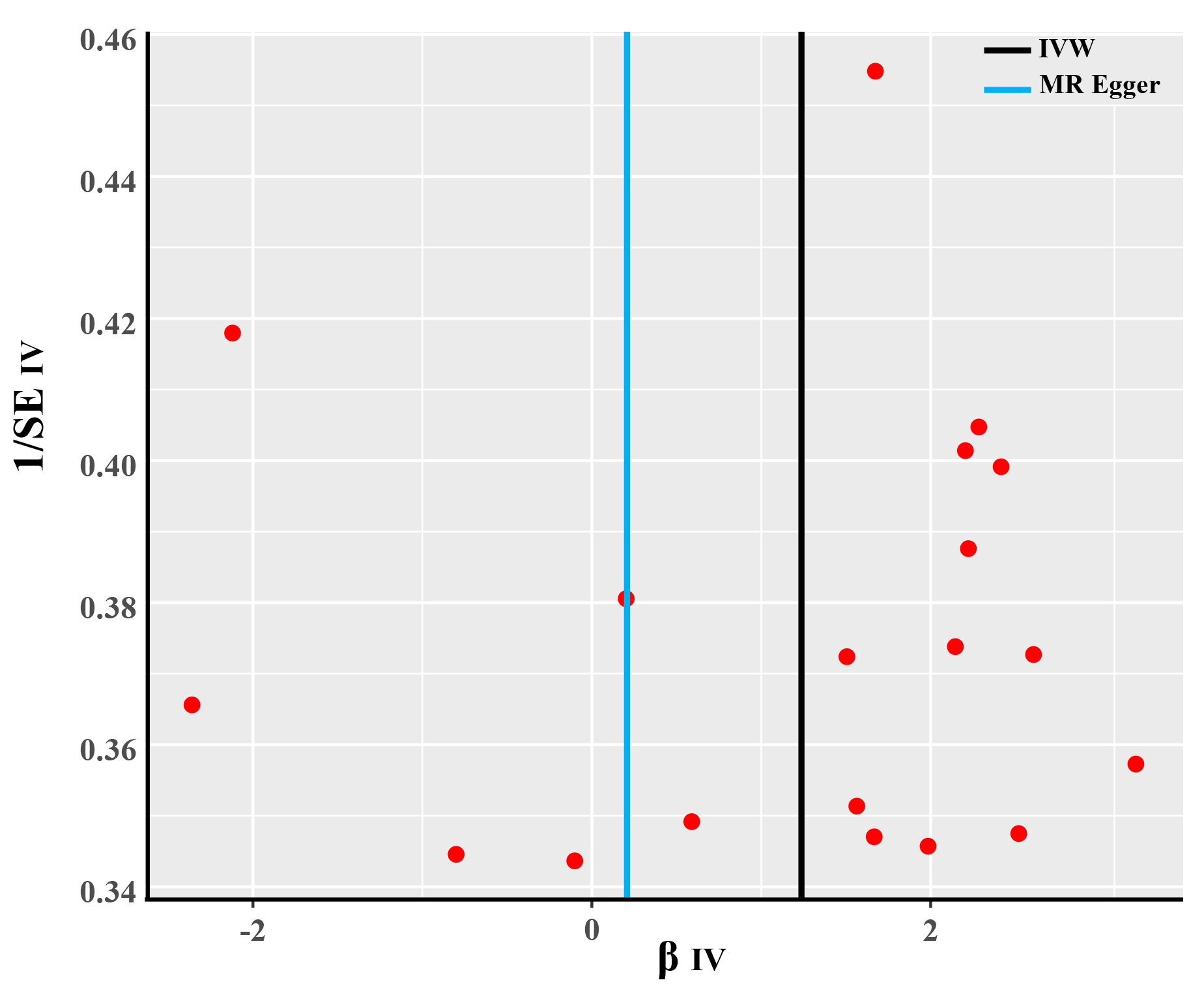

Supplement: Supplementary Figure 1 — The funnel plot in SVMR. IVW, Inverse variance weighted method; SVMR, single-variable mendelian randomization. IV, instrumental variable; SE, standard error; MR, mendelian randomization. [file Image_1.jpeg]

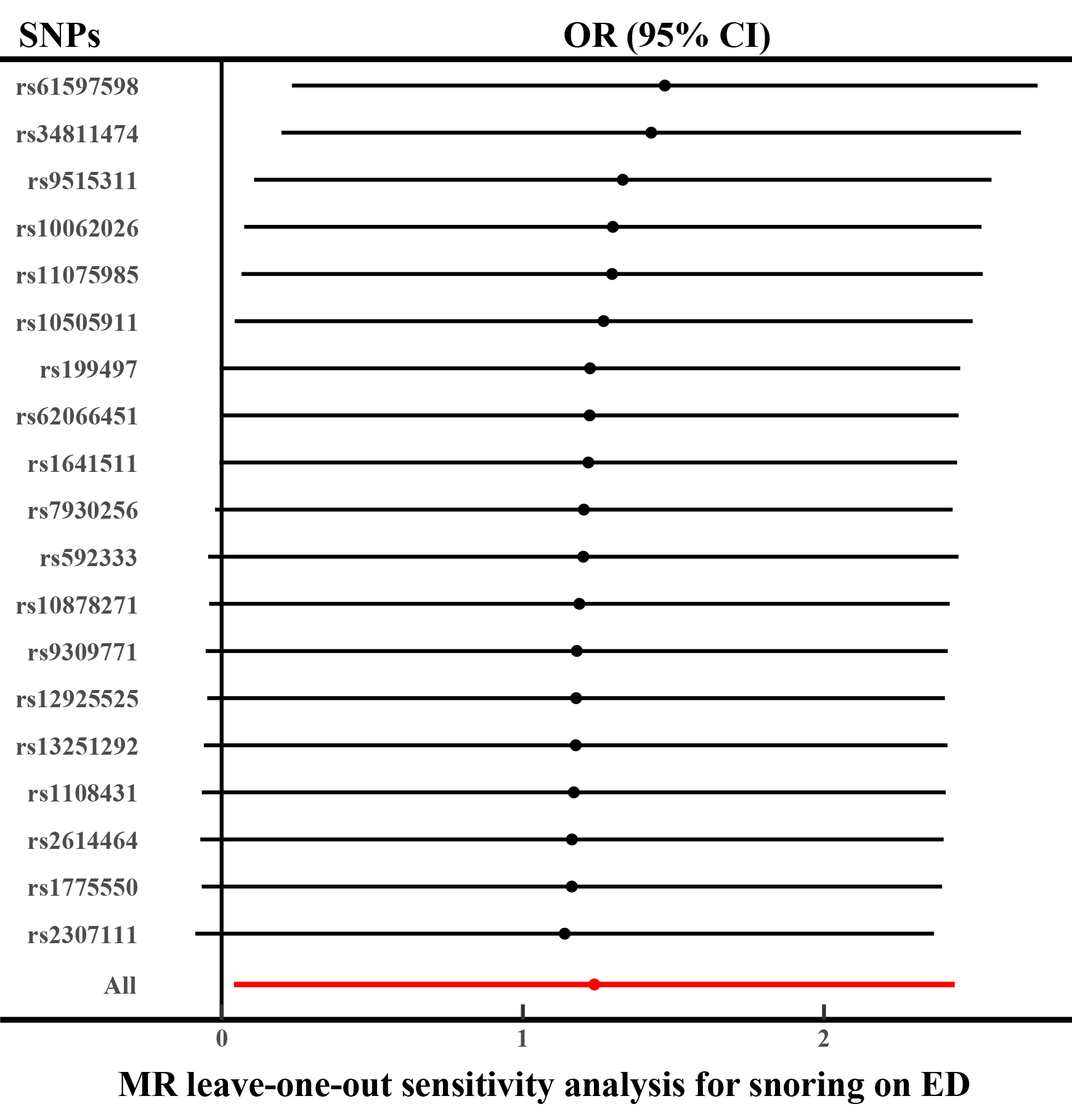

Supplement: Supplementary Figure 2 — The leave-one-out sensitivity analyses of snoring and ED. SNP, single nucleotide polymorphism; OR, odds ratio; CI, confidence interval; ED, erectile dysfunction; MR, mendelian randomization. [file Image_2.jpeg]

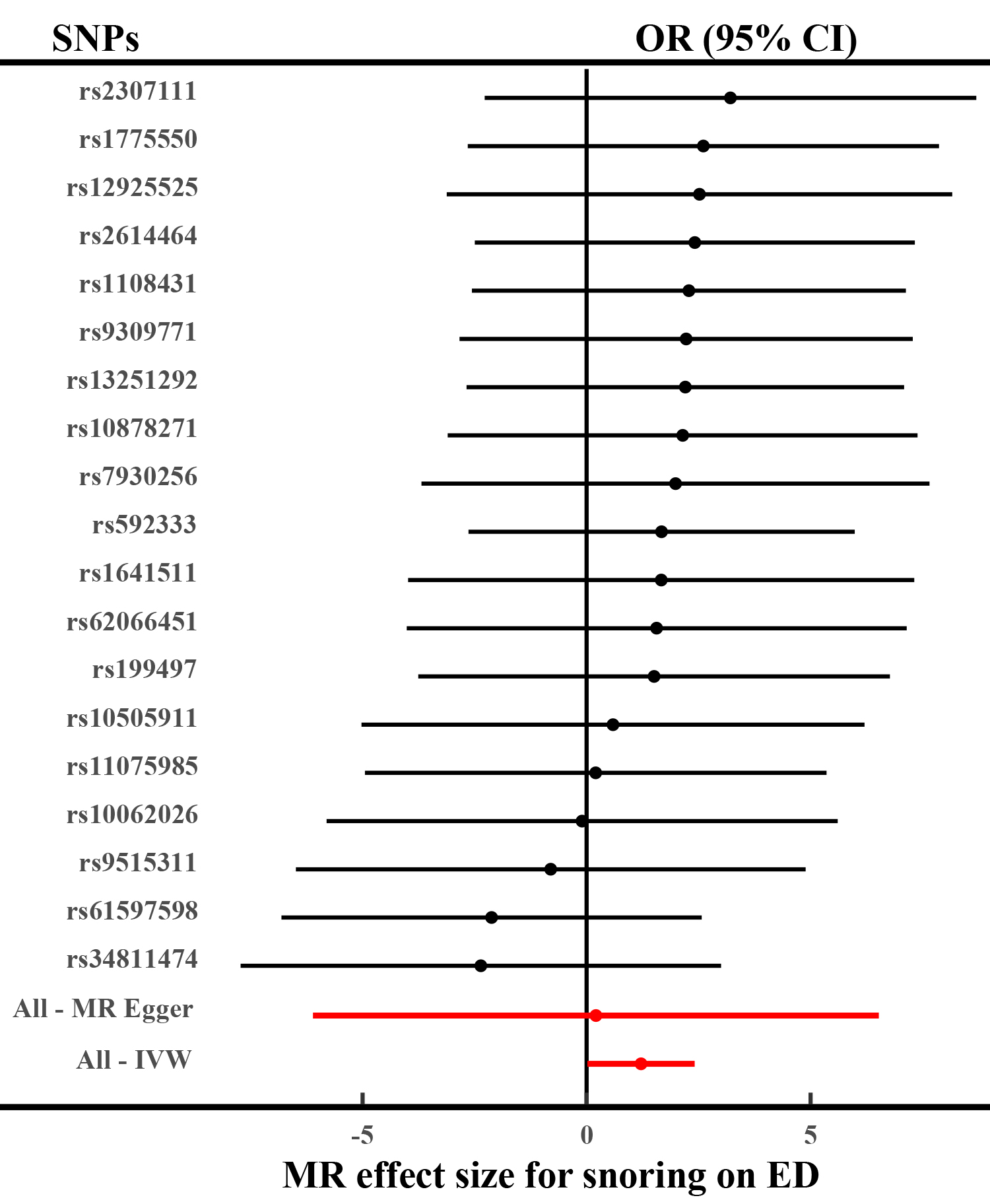

Supplement: Supplementary Figure 3 — The results of MR analyses of causal associations between each snoring SNP and ED. SNP, single nucleotide polymorphism; OR, odds ratio; CI, confidence interval; ED, erectile dysfunction; MR, mendelian randomization. [file Image_3.jpeg]
