# Supplementary material for: Genetic Evidence Supporting a Causal Role of Snoring in Erectile Dysfunction
Source: Front Endocrinol (Lausanne). 2022 May 25;13:896369. doi: 10.3389/fendo.2022.896369 (PMC9174907; doi:10.3389/fendo.2022.896369)
Supplement: Supplementary file 4 [file Table_1.pdf]

Table S1: Data sources of snoring, ED and adjusted covariates

Phenotype	Sample size	Ancestry	Consortium	PMID
**Erectile dysfunction**	223,805 participants (6,175 cases & 217,630 controls)	European	NA	30583798
**Snoring**	314,449 subjects (117,812 cases & 196,637 controls)	European	UK biobank	NA
**Low-density lipoprotein**	188,577 subjects	Mixed	GLGC	24097068
**Total cholesterol**	188,577 subjects	Mixed	GLGC	24097068
**Triglyceride**	188,577 subjects	Mixed	GLGC	24097068
**Cigarette consumption**	1,200,000 subjects	European	GSCAN	30643251

UK: United Kingdom; GLGC: The Global Lipids Genetics Consortium; GASCAN: GWAS and Sequencing Consortium of Alcohol and Nicotine use; PMID: PubMed Identifier
